# Facile Morphology and Porosity Regulation of Zeolite ZSM-5 Mesocrystals with Synergistically Enhanced Catalytic Activity and Shape Selectivity

**DOI:** 10.3390/nano12091601

**Published:** 2022-05-09

**Authors:** Feng Lin, Zhaoqi Ye, Lingtao Kong, Peng Liu, Yahong Zhang, Hongbin Zhang, Yi Tang

**Affiliations:** 1 Department of Chemistry, Shanghai Key Laboratory of Molecular Catalysis and Innovative Materials, and Laboratory of Advanced Materials, Fudan University, Shanghai 200433, China; 20210220046@fudan.edu.cn (F.L.); 17110220035@fudan.edu.cn (Z.Y.); konglingtao@fudan.edu.cn (L.K.); zhangyh@fudan.edu.cn (Y.Z.); 2 Institute for Preservation and Conservation of Chinese Ancient Books, Fudan University Library, Fudan University, Shanghai 200433, China; liupengfdu@fudan.edu.cn

**Keywords:** zeolite ZSM-5, seed-assisted synthesis, mesocrystal, synthesis parameters and crystallization, chemical etching, xylene adsorption/isomerization

## Abstract

The morphology and mesoporosity of zeolite are two vital properties to determine its performance in diverse applications involving adsorption and catalysis; while it remains a big challenge for the synthesis and regulation of zeolites with exceptional morphology/porosity only through inorganic-ions-based modification. Herein, by simply optimizing the alkali metal type (K^+^ or Na^+^), as well as alkali/water ratio and crystallization temperature, the zeolite ZSM-5 mesocrystals with diverse mesostructures are simply and controllably prepared via fine-tuning the crystallization mechanism in an organotemplate-free, ions-mediated seed-assisted system. Moreover, the impacts of these key parameters on the evolution of seed crystals, the development and assembly behavior of aluminosilicate species and the solution-phase process during zeolite crystallization are investigated by means of directional etching in NH_4_F or NaOH solutions. Except for the morphology/mesoporosity modulation, their physical and chemical properties, such as particle size, microporosity, Si/Al ratio and acidity, can be well maintained at a similar level. As such, the *p*/*o*-xylene adsorption and catalytic performance of *o*-xylene isomerization are used to exhaustively evaluate the synergistically enhanced catalytic activity and shape selectivity of the obtained products. This work demonstrates the possibility of effectively constructing novel zeolite mesostructures by simply altering parameters on simple ions-controlled crystallization and provides good models to inspect the impacts of mesoporosity or morphology on their catalytic performances.

## 1. Introduction

As one kind of important inorganic open-framework material, aluminosilicate zeolites are widely used as high-performance adsorbents and catalysts in the fields of petroleum, coal and fine chemicals [1,2,3,4]. For these zeolite materials, their intrinsic microporous networks and abundant tunable acid sites collectively endow them with excellent shape-selective and acid-catalytic properties [5,6,7]. By fine-tuning its morphology, size and structure, the related catalytic properties can be further optimized to increase the reaction rate and conversion, enhance the coke capacity and catalyst stability and modulate the reaction path and product selectivity [8,9,10,11,12]. In recent years, a series of studies have been devoted to directly controlling the morphology and textural properties of zeolite crystals during the crystallization process [13,14,15,16,17]. Among them, the most ideal and basic strategy is to adjust the size and meso-microscopic structure of zeolite particles by simply adjusting the gel compositions and hydrothermal conditions, of which no additional organic templating agents or redundant processing steps are adopted during the synthesis [18,19,20]. However, the regulation based on this idea is often empirical and phenomenological since it is not fully understood for the crystallization mechanism of zeolite and the specific effects of synthesis conditions on its crystallization behavior.

With the continuous in-depth research on the thermodynamics of zeolite crystallization, the specific effects of synthesis parameters on the morphology and crystallization behavior of zeolite have been continuously revealed [21,22,23,24,25,26,27]. For example, Tang and co-workers [22] explored the crystallization mechanism of ZSM-12 zeolites under different water amounts and found the coexistence of molecule/ion mediated growth and crystallization by particle attachment (CPA). For this intertwined classical/nonclassical system, the classical process is predominant for high H_2_O/SiO_2_ ratio to form a denser single-crystal-like structure; while under low H_2_O/SiO_2_, the loose nanocrystallite assemblies are formed because of the dominance of nonclassical process. Yu and co-workers [24] tracked the growth process of silicalite-1 zeolite at different temperatures and found that the nonclassical CPA process was more pronounced at low temperature, resulting in the formation of products with a rough surface; while at high temperature, significant classical growth occurred simultaneously, and the obtained product presented smooth surface and regular shape. Besides this, Valtchev and co-workers [26,27] investigated the crystallization behavior of zeolite L, and it showed that different alkalinities will affect the state and composition of the sol-gel precursors, thereby changing the shape and number of nucleation, and then obtaining products in the form of disc, column or nanoclusters. Therefore, for future development of the simple and ideal synthesis methods, more experimental studies should be devoted to addressing how changes in each synthesis parameter alter the physicochemical state of the system and its corresponding effects on nucleation and/or growth, finally modifying the morphology and properties of zeolites.

As a facile, low-cost and green alternative, the seed-assisted synthesis has recently attracted a lot of research interest, and it can greatly accelerate the nucleation process and even bypass the induction period, in which seeds, as special (alumino)silicate species, can be used as solids for heterogeneous nucleation or as substrates for epitaxial growth [28,29,30,31,32]. Herein, by manipulating the key synthesis parameters, for example, M_2_O/SiO_2_ (M = Na, K), H_2_O/SiO_2_ and crystallization temperature, in a simple ions-mediated seed-assisted system, a series of zeolite ZSM-5 mesocrystals with varied mesostrucutres are synthesized, such as single-crystal-like structure, nanocrystallite assembly and intracrystal mesopore-enriched crystal. Interestingly, by exploring the causal relationships of these parameters, it is found that the zeolite nucleation and growth processes can be facilely tuned by using Na^+^/K^+^ as the simplest and cheapest zeolite growth modifiers (ZGMs). The formation mechanism of diverse ZSM-5 mesocrystals is further deconstructed through selectively post-etching treatment (NaOH and NH_4_F). Because of the similar particle size, microporosity, composition and acidity, the obtained samples with different morphologies/mesoporosity provide a good model to inspect the mesostructure-performance relationship on the diffusion-controlled and shape-selective xylene isomerization and adsorption.

## 2. Materials and Methods

### 2.1. Sample Synthesis

A mixture with a composition of *n*_SiO2_/*n*_Al2O3_/*n*_M2O_/*n*_H2O_ = 1:0.0125:x:y was prepared first with 40 wt% colloidal silica, Al_2_(SO_4_)_3_·18H_2_O, NaOH or KOH and H_2_O (here, M represents K or Na). Then the pre-prepared silicalite-1 seed dispersion was added, and the resulting gel solution was aged at room temperature for a certain period (normally 3 h). Next, the gel was moved into the autoclave and heated up to a higher temperature (*T*/°C) for a hydrothermal period to prepare the zeolite product denoted as M-Z5-x-y-*T*. The solid products were then recovered by filtration and washed with deionized water. The silicalite-1 zeolite with a size of ca. 250 nm was used as seeds and the quantity of the pre-added seeds typically equaled 7.0 wt% of the total SiO_2_ weight in the starting gel. The silicalite-1 seeds were synthesized by a clear solution method [33], and the obtained suspension was directly used as seed without further treatment.

To investigate the impacts of key crystallization factors in this K^+^/Na^+^ ions-mediated seed-assisted system, a series of products with different morphologies were prepared under different H_2_O/SiO_2_ ratios, M_2_O/SiO_2_ ratios and crystallization temperatures with/without silicalite-1 seeds. The detailed synthetic conditions are listed in Table 1. In order to ensure the completion of crystallization and avoid the probable crystal transformation and the generation of other impurities during a too-long hydrothermal time, the hydrothermal synthetic time of each sample is shown in Table 1. The catalytic control sample SC-Na-Z5 was synthesized by the traditional seed crystal route at 140 °C, of which the feeding ratio in the initial gel was changed to *n*_SiO2_/*n*_Al2O3_/*n*_M2O_/*n*_H2O_ = 100:1.25:28:8000. In order to further obtain H^+^-form ZSM-5, the calcined M-Z5 was ion-exchanged three times with 5 wt% NH_4_NO_3_ solution at 90 °C and then calcined at 550 °C for 6 h.

The calcined ZSM-5 zeolite was dispersed in 25 or 40 wt% NH_4_F aqueous solutions with a solid/liquid ratio of 1/30 and then reacted at 50 °C for 30 min. The treatment of 2 wt% NaOH was carried on with 1 g of the calcined ZSM-5 zeolite dispersed in 40 g of solution and then treated at 80 °C for 60 min.

### 2.2. Sample Characterizations

The powder X-ray diffraction (XRD) experiments were carried out on a Bruker D2 diffractometer (Cu-Kα, 10 kV, 30 mA, Bruker, Karlsruhe, Germany) from 5 to 35° to judge the framework type of obtained products. Scanning electron microscopy (SEM, Phenom Prox, Phenom, Eindhoven, Netherlands) and field-emission scanning electron microscopy (FE-SEM, Hitachi S-4800, Hitachi, Tokyo, Japan) were employed to observe the morphologies of the samples. Field-emission transmission electron microscope (FE-TEM, Tecnai G2 F20 S-Twin, FEI Inc., Hillsboro, OR, USA) was applied to access the structural feature. The textural parameters of the calcined products were measured on a Quantachrome iQ-2 instrument (Quantachrome, Boynton Beach, FL, USA) by N_2_ sorption experiments at 77 K after outgassing at 573 K for 6 h. The BET surface areas are determined by the relative pressure *P*/*P*_0_ from 0.005 to 0.035 according to the Rouquerol criterion. The status of aluminum in zeolite was obtained by solid-state ^27^Al MAS NMR (Bruker DSX 300, Bruker, Karlsruhe, Germany). The acidic properties of typical samples were obtained by NH_3_ temperature-programmed desorption (NH_3_-TPD) experiment on the Micromeritics AutoChem II 2920 (Micromeritics, Norcross, GA, USA). About 0.1 g of 40–60 mesh sample was activated under He flow at 550 °C for 60 min and then cooled to 80 °C. Then, the sample was thermostatically adsorbed in a 10 vol% NH_3_-He flow for 1 h followed by a He purge to remove the physisorption. Finally, the sample was desorbed at 10 °C·min^−1^, and the desorption signal was recorded with a TCD detector. Fourier transform infrared (FT-IR) spectroscopy measurements were performed by a Nicolet 6700 spectrometer (Thermo Fisher Scientific, Waltham, MA, USA). The samples were pressed into thin wafers and were degassed in a vacuum cell for 120 min at 450 °C. Then, the sample was cooled down to room temperature and a background spectrum was recorded. Next, consecutive doses of pyridine were added to the sample until saturation. Finally, the sample was heated to 300 °C under a vacuum environment for 30 min, and the spectra were then recorded.

### 2.3. Adsorption and Catalytic Reactions

The measurement of *p*/*o*-xylene adsorption on typical ZSM-5 samples was performed using a computer-controlled intelligent gravimetric analyzer (IGA, Hiden Analytical Ltd., Warrington, UK). First, the sample (10 mg) was degassed under a vacuum of less than 10^−3^ Pa at 673 K for 2 h, and the adsorption measurement (298 K) was performed after the sample cooling to ambient temperature (keeping vacuum). Subsequently, the gaseous adsorbate (such as *p*-xylene vapor) was injected in pulse mode to the pre-set relative pressure under the control of the computer. The adsorption kinetic curve was recorded by the intelligent precise balance until the mass was almost unchanged, and the instrument automatically adjusted to the next pressure to start adsorption.

The gaseous phase *o*-xylene isomerization was conducted in a micro-fixed bed reactor (Tianjin Golden Eagle Technology Co., Ltd., Tianjin, China), of which inner diameter is 8 mm. The reaction condition was 673 K under atmospheric pressure. In detail, 0.5 g catalyst (20–40 mesh) was pre-heated at 673 K (1.0 h) prior to isomerization under a N_2_ carrier flowing at 100 mL·min^−1^. During the reaction, *o*-xylene was injected into the vaporization chamber by a metering pump with a 5.0 h^−1^ weight hourly space velocity (WHSV), and the N_2_ carrier flow rate was 40 mL·min^−1^. Moreover, the products were analyzed per 30 min using an online GC equipped with a flame ionization detector (FID) and a high-solution capillary column (HP-INNOWAX).

## 3. Results and Discussion

### 3.1. Optimal Condition Investigation for the Synthesis of ZSM-5 Mesocrystals

The influences of the most important factors on the morphology were firstly investigated under this simple ions-mediated seed-assisted hydrothermal crystallization process, such as ion type (Na^+^ or K^+^), H_2_O/SiO_2_ ratio (water amount), M_2_O/SiO_2_ ratio (alkalinity) and hydrothermal temperature. The detailed synthesis conditions and product phases involved in this work are listed in Table 1.

#### 3.1.1. The Influence of the H_2_O/SiO_2_ Ratio 

**Figure 1:**
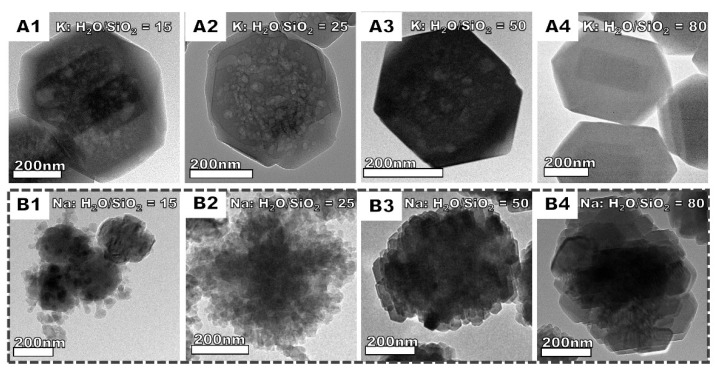
The TEM images of the products synthesized in the K^+^ system with H_2_O/SiO_2_ = (**A1**) 15, (**A2**) 25, (**A3**) 50, (**A4**) 80; and the Na^+^ system with H_2_O/SiO_2_ = (**B1**) 15, (**B2**) 25, (**B3**) 50, (**B4**) 80.

Firstly, the effects of the H_2_O/SiO_2_ ratio on the final products are investigated. For the K^+^ system (K-Z5-0.15-y-140, y = 15/25/50/80), their XRD patterns (Figure S1) show that all obtained products are pure MFI phase in the wide range of H_2_O/SiO_2_ = 15–80. Meanwhile, as shown in SEM images (Figure S2), all samples possess relatively smooth surfaces and similar particle sizes in the range of 450–600 nm. TEM results (Figure 1(A1-4)) further present the differences between them. Specifically, in the inner part of samples (Figure 1(A1,A2)) exists abundant intracrystal mesopores with a size of about 20–40 nm when the H_2_O/SiO_2_ is controlled at 15 or 25. However, the amount of intracrystal mesopores significantly decreases at H_2_O/SiO_2_ = 50 (Figure 1(A3)), and the zeolite ultimately exhibits dense structure without obvious intracrystal mesopores when the H_2_O/SiO_2_ reaches 80 (Figure 1(A4)).

As for the Na^+^ system (Na-Z5-0.15-y-140, y = 15/25/50/80), almost all samples are from the pure MFI phase (see XRD results in Figure S3), except for the sample of H_2_O/SiO_2_ = 15 containing a small amount of impure phase (2θ = 5.5°). The TEM and SEM results (Figure 1(B1–4) and Figure S4) show that the nanocrystal assembled structure can be gradually formed on the seed surface when H_2_O/SiO_2_ is increased from 15 to 25. Especially, the sample of H_2_O/SiO_2_ = 25 exhibits the loosest nanocrystallites stacking structure with nanocrystallite domains even less than 20–30 nm. When H_2_O/SiO_2_ increases to 50 and 80 (Figure 1(B3,B4)), these nanocrystallites tend to bridge and fuse together to be densified and the size of these domains distinctly increases to 50–80 nm and 100–200 nm, respectively, and the edges of the whole assembly also become more regular and sharper. Compared with traditional synthetic methods that tune the morphology with the help of organic templating agents or complex post-processing procedures [3,8], we finely control the morphology of zeolite by simply altering H_2_O/SiO_2_ in an ion-mediated seed-assisted system, which obtains either the ZSM-5 zeolite with diverse sizes and amount of intracrystal mesopores or the nanocrystallite assembly with the varied size and amount of opened mesopores due to the various-sized nanocrystallite.

#### 3.1.2. The Influence of the M_2_O/SiO_2_ Ratio 

**Figure 2:**
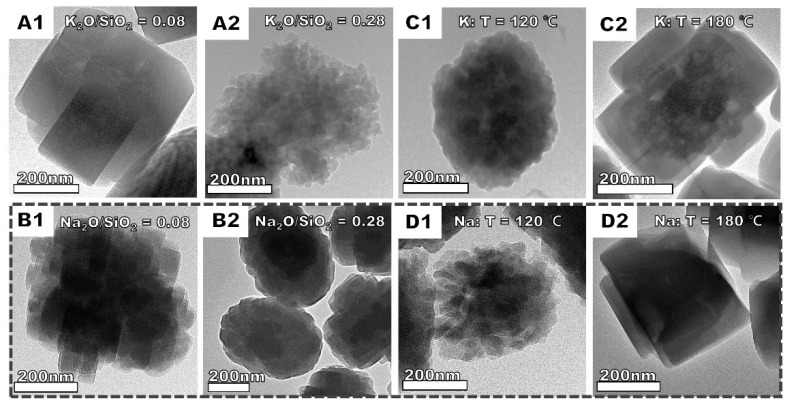
The TEM images of the products synthesized in the K^+^ system with M_2_O/SiO_2_ = (**A1**) 0.08, (**A2**) 0.28; the Na+ system with M2O/SiO2 = (**B1**) 0.08, (**B2**) 0.28; the K+ system at hydrothermal temperature of (**C1**) 120 °C, (**C2**) 180 °C; and the Na+ system at hydrothermal temperature of (**D1**) 120 °C, (**D2**) 180 °C.

Next, it is also investigated the impacts of the alkalinity on the product morphologies. As shown in Figure S5, for both K^+^ and Na^+^-mediated seed-assisted systems (K-Z5-x-25-140 and Na-Z5-x-25-140), all products are pure MFI zeolite in the studied alkalinity range (x = 0.08/0.15/0.28). When the K_2_O/SiO_2_ of K-Z5-x-25-140 samples drops from 0.15 to 0.08, the obtained products keep the smooth crystal surface (Figure 2(A1)), but the amount of intracrystal mesopores sharply decreases. While the K_2_O/SiO_2_ is raised to 0.28, the products transform into the nanocrystallite assembled structure (Figure 2(A2)). Although the morphology is similar to that of Na-Z5-0.15-y-140 (mainly for y = 25/50), it seems that the core (i.e., the parent silicalite-1 seed) inside the product crystal is not well-preserved and probably dissolved, considering the low contrast and loose structure of the central region shown in the TEM image. In regard to the Na^+^ system, at a lower alkalinity of Na_2_O/SiO_2_ = 0.08, the structure of nanocrystallite assembly is basically maintained (Figure 2(B1)). Otherwise, with the increase of alkalinity to Na_2_O/SiO_2_ = 0.28, the obtained products transform into compact assembled crystals with much larger-sized nanocrystal domains (Figure 2(B2)).

#### 3.1.3. The Influence of the Hydrothermal Temperature 

Hydrothermal temperature is another most impactful parameter. It has been found that the low temperature favors nucleation over crystal growth, while the high temperature tends to facilitate the addition of simple aluminosilicate species [13]. Thereby, as shown in Figure 2(C1,D1), the low hydrothermal temperature of 120 °C contributes to the formation of nanocrystallite assembled structures for both K^+^ and Na^+^-mediated systems. When the temperature increases to 180 °C, these two systems all tend to form dense structures with more regular and sharper edges (Figure 2(C2,D2)). However, different from the solid dense structure of the Na^+^-mediated system, the K^+^-mediated products show a hollow structure with enriched intracrystal mesopores.

### 3.2. Etching Treatment and Understanding of the Formation Processes

**Scheme 1:**
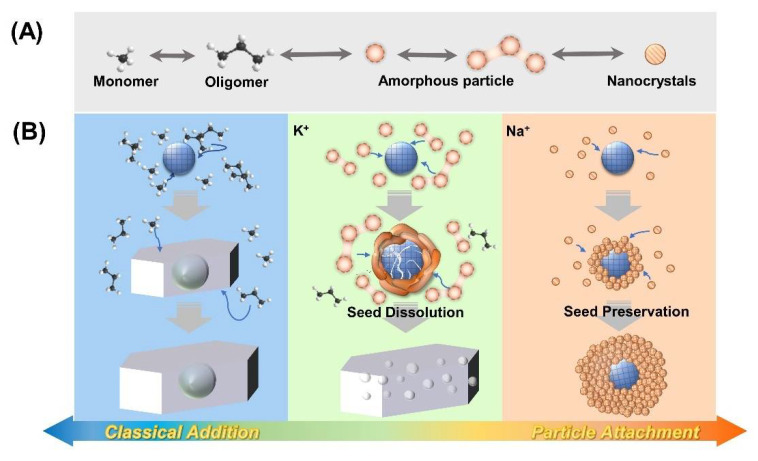
(**A**) The evolutions of diverse crystal-grown nutrients. (**B**) The possible crystallization pathways in K^+^/Na^+^-mediated seed-assisted systems.

The morphologies and textural properties of zeolites are intimately related to their crystallization process and mechanism. During the crystallization, the crystal-grown nutrients can be added to the crystal nuclei in the form of monomers/oligomers (i.e., classical crystallization) [34,35] or transformed first into various precursor particulate species illustrated in Scheme 1A with metastable and evolving properties (e.g., different size and shape, solid- or liquid-like state, amorphous or crystalline) and then they can undergo crystallization by particle attachment (CPA, i.e., nonclassical crystallization) [36,37,38,39]. Meanwhile, in the process of seed-assisted synthesis, the seed crystals are added to a reaction mixture and can be used as substrates for epitaxial growth or as special aluminosilicate species for heterogeneous nucleation [39,40,41], as displayed in Scheme 1B. In this work, the seed-induced interfacial crystallization and intertwined classical/nonclassical process are likely to occur together, so that a series of the above-mentioned ZSM-5 mesocrystals were harvested with diverse internal inhomogeneities and porous architectures.

Recently, selectively chemical post-treatment of the matured zeolite crystals has been developed to provide new information and a deeper understanding of zeolite crystallization and related nano-scale architecture [42,43,44,45,46]. Here, to gain the inner structural details and reveal the underlying formation mechanisms, two typical samples (K-Z5-0.15-25-140 and Na-Z5-0.15-25-140) were treated by the directional etching experiments of NH_4_F (25 wt%, 40 wt%) and NaOH (2 wt%) solutions. The former unbiasedly etches both Si and Al in the framework, which could reflect the defect-rich region inside the zeolite [42,43,44]; while the latter mainly removes silicon-rich parts in zeolite, which could indicate elemental inhomogeneous distribution in zeolite crystals [45,46]. Meanwhile, the relationship between the synthesis parameters/conditions and the possible crystallization mechanisms is further comprehensively understood.

#### 3.2.1. Effect and Evolution of Seed Crystals

**Figure 3:**
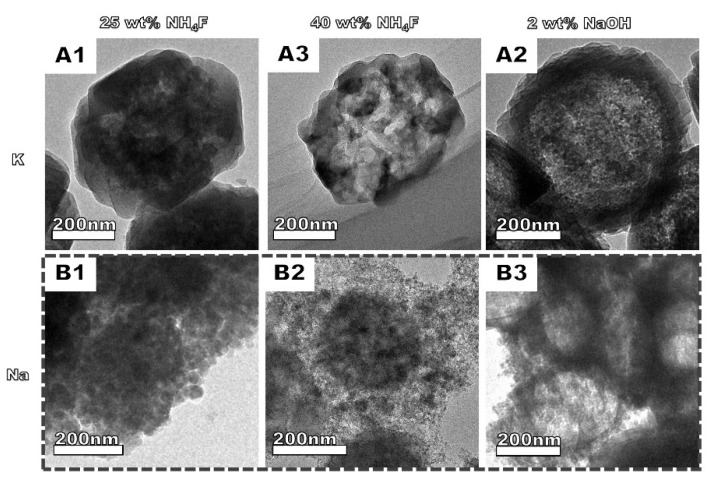
The TEM images of K-Z5-0.15-25-140 products etched via (**A1**) 25 wt% NH_4_F, (**A2**) 40 wt% NH_4_F, (**A3**) 2 wt% NaOH solutions; and Na-Z5-0.15-25-140 products etched via (**B1**) 25 wt% NH_4_F, (**B2**) 40 wt% NH_4_F, (**B3**) 2 wt% NaOH solutions.

Through NH_4_F post-treatment from low to high concentration (Figure 3(A1,A2)), the internal mesopore wall of K-Z5 zeolite is gradually etched, causing the original intracrystal mesopore (10–20 nm) connecting each other to form irregular pores of about 50–100 nm, which are randomly distributed in zeolite without complete seed crystals inside. Combined with the parent K-Z5 product morphologies (Figure 1(A2), with a relatively empty structure in the crystal center), we can infer that K^+^ ions play significant roles in the “sacrifice” (i.e., dissolution) of the seed and subsequent crystallization of K-Z5 zeolite, as illustrated in K^+^ system of Scheme 1B. Comparatively, a spherical cavity (ca. 400 nm) appears in K-Z5 zeolite after NaOH etching (Figure 3(A3)), which is much larger than the seed size (ca. 250 nm), implying that the siliceous seeds may easily sacrifice and transform into the nutrients integrating with original stacked Si-Al species in the precursor particles to lead a Si-rich zone in the inner region of final crystals.

As for the Na^+^ system, the fate of seeds in the zeolite is completely different and they are likely to be preserved. As shown in Figure 3(B2) after NH_4_F etching, the inner seeds (spherical cores of ca. 250 nm) are well-maintained but with partial destruction of the outer crystallite shell, suggesting that Na^+^ ion has no obvious damage to seeds during zeolite crystallization compared with K^+^. In addition, different from the K^+^-mediated system, the NaOH etching on Na^+^-mediated products leads to a hollow center of the same size as the silicalite-1 seed (Figure 3(B3)), further demonstrating the core–shell assembled structure of the product formed via epitaxial growth of aluminosilicate species on the well-preserved seed, as shown in Na^+^ system of Scheme 1B.

Besides this, no matter whether the seeds are sacrificed or preserved (K^+^ or Na^+^ system), the introduction of seeds greatly accelerates the crystallization rate. The controlled experiments of Na^+^/K^+^ systems without seeds (Figure S7) show that products still have an amorphous phase even after 72 h crystallization, especially for K-Z5 with no MFI structure. However, it should be noted that the crystallization rate of the K^+^ system (<24 h) is much faster than that of the Na^+^ system (<72 h) after seed addition. According to the previous literature and results, this phenomenon may be attributed to the fact that the sacrificed crystal seeds will produce abundant structural species, especially the structural species with the “memory” of the original seeds [40,47,48] or alter the local supersaturation, which will be very conducive to the zeolite crystallization.

#### 3.2.2. Development and Assembly Behavior of Aluminosilicate Particles

After selective etching of the boundaries and defect-rich regions of crystals by NH_4_F, the original coffin-shape and smooth surfaces of K-Z5 gradually become irregular and undulate (Figure 3(A2)). And interestingly, they present an obvious stacking structure of several large-sized “worm-like” domains. Further observation shows that the outer “worm-like” crystal domains are somewhat larger than those in the inner region of zeolites, implying the evolution of these attached precursor particles during crystallization. The NaOH etching results (Figure 3(A3)) also reveal the differences between internal and external “worm-like” particles. The inner of K-Z5 is completely dissolved, while the outer layer is preserved well, which indicates the gradient Al-zoning in K-Z5 and the lower Si/Al ratio in the shell. Accordingly, it can be speculated that the “worm-like” particles first attached to the seed surfaces will crosslink with siliceous species dissolved from silicalite-1 seed and then crystallize together with them, leading to a gradient Si/Al ratio distribution in the final zeolite crystals, as shown in the K^+^ system of Scheme 1B.

For the Na^+^ system, after NH_4_F treatment, the nanoparticles-assembled structure on seed crystals is exhibited more clearly (Figure 3(B1,B2)). Such etching phenomenon implies that in the Na^+^ system, the nanocrystallites act as building units and assemble on the seed surface to form the nanocluster products. The synthesis results with different water amounts support this inference. As the H_2_O/SiO_2_ increases from 25 to 80, the nanocrystallite units become larger, resulting in a corresponding increase in the domain size in the final products. Serendipitously, when the H_2_O/SiO_2_ equals 15, the initial attachment of nanocrystallites on the seed surfaces can be clearly observed, though the other aluminosilicate species form impure phases due to inappropriate growth conditions. In addition, compared with the seed-free K^+^ system, the MFI-structured crystals can still be formed in the unseeded Na^+^ system (Figure S7), suggesting the spontaneous nucleation capability of the Na^+^-mediated precursors. As such, the nanoparticles in the Na^+^ system undergo self-nucleation and then grow onto the seeds by the oriented interfacial assembly process to construct the final cluster products (Scheme 1B).

#### 3.2.3. Solution-Phase Process (Classical Crystallization Process)

Apart from the CPA processes, the products’ morphologies and the etching results indicate that the monomer/oligomer addition process (classical crystallization) also takes part in the growth of these ZSM-5 mesocrystals. For K-Z5, the results of NH_4_F etching show that the outer regions of zeolite crystals have better thermodynamic stability and more perfect structure than the inner parts since the deconstruction starts from the inside areas first (Figure 3(A1)). Meanwhile, the original K-Z5-0.15-25-140 sample (Figure S2B) has a coffin-shaped regular morphology and smooth surface, which implies the non-negligible role of classical crystallization in zeolite formation. Generally, the remaining aluminosilicates in the solution phase (i.e., the monomers/oligomers) tend to add on the steps/kinks on the crystal surface or repair the crystal defects to achieve a smooth surface and thermodynamically stable structure, especially in the later stage of the crystal growth [36].

Yet, for the Na-Z5-0.15-25-140 sample, the outer surface is rather rough, and the NH_4_F-etching result reveals that the outer layers are defect-rich areas, which can be attributed to the relatively fewer classical crystallization processes in the Na^+^ system. Additionally, the previous synthesis results with different water amounts have shown that all the K-Z5 samples maintain the smooth surface features (Figure S2), while the surfaces of Na-Z5 products change from rough to smooth gradually with the increase in H_2_O/SiO_2_ (Figure S4). The different morphological drift trends between the two sequences indicate that there is a more pronounced liquid-phase monomer/oligomer addition in the K^+^ system, regardless of how concentrated or diluted it is (Scheme 1B). Whereas for the Na^+^ system, the classical crystallization process plays a significant role only at a high H_2_O/SiO_2_ ratio.

### 3.3. Characterization of Porous Structure and Acid Property

**Figure 4:**
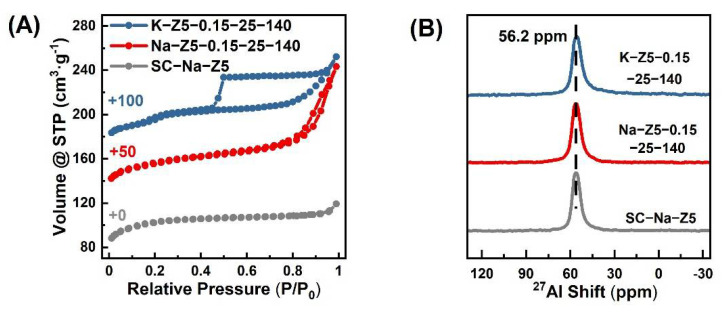
(**A**) N_2_ sorption isotherms at 77 K and (**B**) ^27^Al MAS NMR spectra over three typical ZSM-5 zeolite samples.

The properties of three typical ZSM-5 mesocrystals are further investigated, including the single-crystal-like sample (SC-Na-Z5, of which the TEM image is shown in Figure S8), the nanocrystallite assembly (Na-Z5-0.15-25-140) and the intracrystal mesopore-enriched crystal (K-Z5-0.15-25-140). The differences in their pore structures are evidenced by N_2_ sorption experiments (Figure 4A). Both K-Z5-0.15-25-140 and Na-Z5-0.15-25-140 show type I and IV isotherms but with different shaped hysteresis loops, indicating their distinctive mesopore structures. In detail, the steep increases of all samples at a low relative pressure (*P*/*P*_0_ < 0.1) imply their perfect microporosity. At intermediate and high relative pressure ranges (*P*/*P*_0_ = 0.4–1.0), K-Z5-0.15-25-140 possesses an H2-type hysteresis loop with a characteristic step down on the desorption branch at around *P*/*P*_0_ = 0.43, which can be ascribed to the cavitation effect of the “ink-bottle” type intracrystal mesopores [49]. The observation indicates that most mesopores are occluded within the zeolite crystals, in conformity to TEM images (Figure 1(A2)). Differently, Na-Z5-0.15-25-140 shows a tiny H3-type hysteresis loop with a continuously enhanced adsorption at high pressures (0.75 < *P*/*P*_0_ < 1) but without the characteristic step down at around *P*/*P*_0_ = 0.43 on the desorption branch, which demonstrates the intercrystallite mesopores formed by the assembly of adjacent nanocrystallites is open [11,49], in line with the observation in TEM images (Figure 1(B2)). The pore size distribution and textural properties are shown in Figure S9 and Table S1, respectively. All samples have a large specific surface area and micropore volume [50], indicating their high crystallinity. K-Z5-0.15-25-140 possesses a large mesopore volume (*V*_meso_, ca. 0.149 cm^3^·g^−1^) but a small external surface area (*S*_ext_, ca. 63 m^2^·g^−1^); while Na-Z5-0.15-25-140 possess a large mesopore volume (*V*_meso_, ca. 0.180 cm^3^·g^−1^) and a large external surface area (*S*_meso_, ca. 98 m^2^·g^−1^). These results well verify their different mesopore architectures.

**Figure 5:**
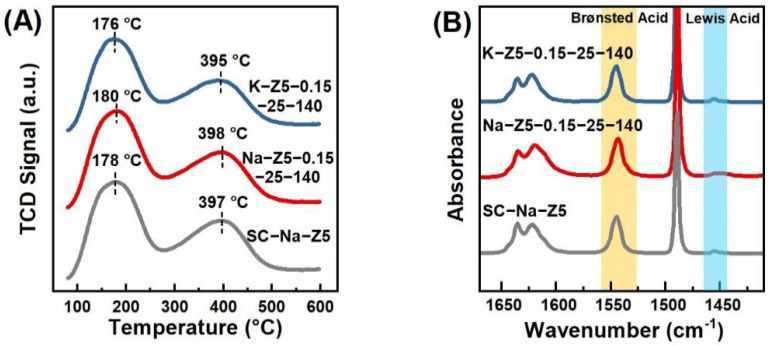
(**A**) NH_3_-TPD profiles and (**B**) FT-IR spectra with pyridine adsorption for three typical ZSM-5 zeolite samples.

^27^Al MAS NMR, NH_3_-TPD and FT-IR are adopted to explore the acid properties of these three samples. The results of ^27^Al MAS NMR are illustrated in Figure 4B and all samples exhibit a sole resonance at 56.2 ppm, which indicates the Al atoms are all tetrahedral coordinated. Then, NH_3_-TPD is applied to investigate the amount and strength of acid sites. As shown in Figure 5A, these three samples all display similar two-peak curves in which the temperature Peak I at about 180 °C and Peak II at about 395 °C are assigned to the weak and strong acid sites, respectively, and the acid amounts of Peaks I or II calculated from the NH_3_ desorption are similar for all three samples, as shown in Table S2, indicating their similar acid site amount and strength distribution. 

Besides this, the Brønsted and Lewis acidity are further studied by FT-IR spectroscopy with pyridine adsorption. As shown in Figure 5B, all the samples exhibit two main bands, that is, 1545 cm^−1^ corresponding to the band of pyridine protonated by Brønsted acid sites (pyridinium ions), 1455 cm^−1^ resulting from the band of pyridine coordinated to the Lewis acid sites. It is noted that the band intensity at 1455 cm^−1^ is much weaker than that at 1545 cm^−1^, and no obvious difference in band intensity at 1545 and 1455 cm^−1^ can be found for these ZSM-5 samples with similar Si/Al ratios but different mesoporous structures. 

### 3.4. Properties of o-Xylene Isomerization Reaction and o/p-Xylene Adsorption

Mixed xylene isomerization to produce *p*-xylene is an important industrial application for zeolites. Among these isomers, pure *o*- or *m*-xylene isomerization is a model reaction to assess the shape selectivity of microporous zeolites [51,52]. Herein, *o*-xylene isomerization and *o*/*p*-xylene adsorption were investigated on these three typical ZSM-5 mesocrystals. Moreover, for clearly investigating the effects of different mesoporous structures on the catalysis and adsorption performance, it is truly significant to modulate these ZSM-5 zeolite mesocrystals with similar characteristics, including intrinsic microporosity, particle size, framework crystallinity and acidity.

**Figure 6:**
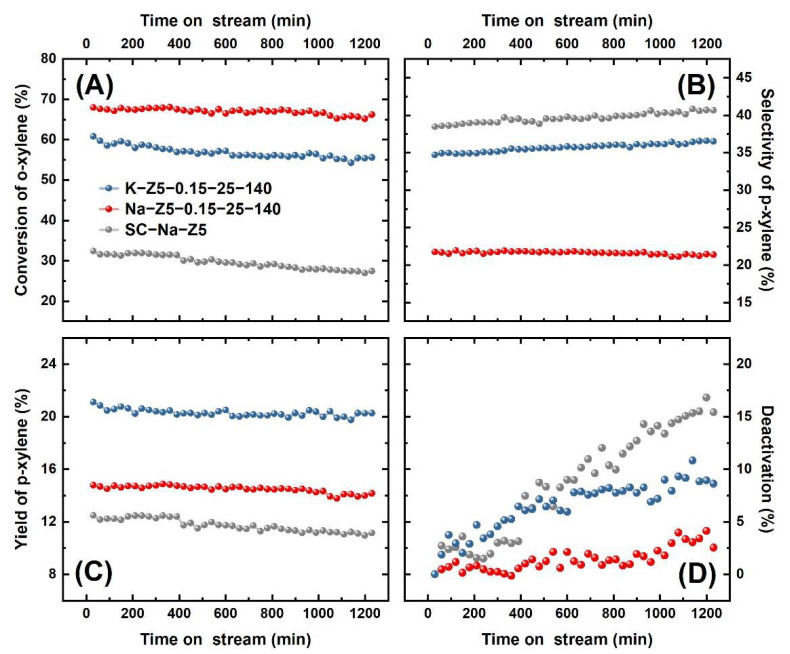
(**A**) Conversion of *o*-xylene, (**B**) selectivity of *p*-xylene, (**C**) yield of *p*-xylene and (**D**) deactivation of catalysts over three typical ZSM-5 mesocrystals. The deactivation is defined as (X_0_ − X)/X_0_, where X_0_ is the initial *o*-xylene conversion and X is the *o*-xylene conversion at time t.

The catalytic results of *o*-xylene isomerization (conversion, selectivity, yield and stability) are shown in Figure 6, which were acquired on a fixed-bed reactor equipped with an online GC for product analysis. As shown in Figure 6A, the *o*-xylene conversions display the results of Na-Z5-0.15-25-140 (ca. 66%) > K-Z5-0.15-25-140 (ca. 59%) >> SC-Na-Z5 (ca. 31%) during the whole reaction run. This should be attributed to the reduced diffusion resistance of the samples with hierarchically porous structures. The existence of mesopores shortens the length of the micropore channel and makes the active sites more efficiently catalyze the reaction of *o*-xylene. Therefore, the smaller size of crystallite and the shorter micropore channel endow the nanocrystallite assembly with a higher *o*-xylene conversion than that of intracrystal mesoporous structure.

The detailed product distribution analysis implies that the main components in the product mixture are the xylenes of three *p*/*o*/*m*-isomers and almost no disproportionation reaction occurs. Interestingly, although with super activity, the *p*-xylene selectivity of K-Z5-0.15-25-140 (ca. 37%, Figure 6B) is only slightly lower than that of perfect single-crystalline SC-Na-Z5 (ca. 40%). While the product selectivity to *p*-xylene (i.e., shape selectivity) of Na-Z5-0.15-25-140 is greatly reduced to ca. 22%. The phenomena can be explained by their special differences in mesoscopic structures. For K-Z5-0.15-25-140, the abundant mesopores are occluded in a well-crystallized boundary shell (thickness of ca. 80 nm) with enriched micropores, so that the number of acid sites on the external surface should be similar to that of SC-Na-Z5. Therefore, the para-selectivity of K-Z5-0.15-25-140 is well maintained in the favor of monomolecular reaction within the microporous network. However, for Na-Z5-0.15-25-140, more external surface area with acid sites will greatly increase the probability of the subsequent secondary reactions, such as the *p*-xylene isomerization to *o*/*m*-xylene and even xylene intermolecular reaction.

Overall, K-Z5-0.15-25-140 displayed a much higher *p*-xylene yield (ca. 22%, in Figure 6C), while Na-Z5-0.15-25-140 showed a smaller increase in *p*-xylene yield (ca. 15%) compared to the pure microporous SC-Na-Z5 (ca. 11%). Besides this, the acid sites located at the external surface without the micropore confinement would trigger the unwanted reaction, such as the formation of the coke, which often blocks the micropores and leads to the deactivation of the catalyst [51,52]. Na-Z5-0.15-25-140 possesses more subsequent secondary reactions than other samples and produces more coke deposits. However, after comparatively analyzing the deactivation of catalysts, it is noted that the stability of *o*-xylene isomerization follows the order of Na-Z5-0.15-25-140 > K-Z5-0.15-25-140 > SC-Na-Z5 (Figure 6D), just in accordance with the trend of *S*_ext_. It indicates that the existence of the mesopores is beneficial to the improvement of coke tolerance capability, especially in the case of the open mesopores (Na-Z5-0.15-25-140).

**Figure 7:**
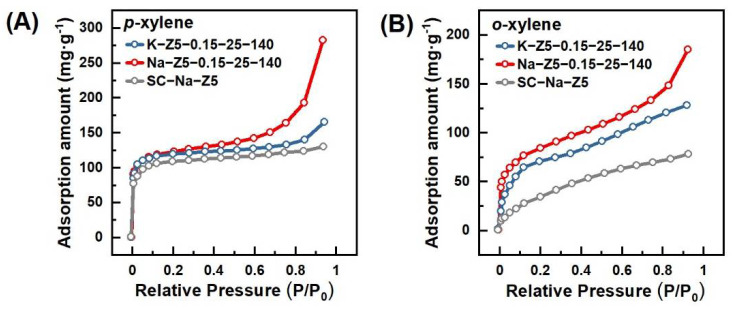
(**A**) The *p*-xylene adsorption isotherms of different ZSM-5 mesocrystals by IGA at 298 K, (**B**) The *o*-xylene adsorption isotherms of different ZSM-5 mesocrystals by IGA at 298 K.

In order to explore the detailed transport properties of both reactants and products on these hierarchical ZSM-5 mesocrystals, their adsorption and diffusion properties are evaluated by studying the gravimetric uptake of *p*-xylene (0.58 nm) or *o*-xylene (0.68 nm) on an IGA apparatus (Figure 7). It is known that *p*-xylene can facilely enter the micropore channels of MFI zeolites (0.55 nm) due to their similar sizes, whereas the diffusion of bulkier *o*-xylene is hindered so that it mainly binds to the external surface or the micropore mouths of the catalyst [30,31,53]. For *p*-xylene, all three samples show high equilibrium adsorption capacity at low pressure (*P*/*P*_0_ < 0.1), which corresponds to the adsorption in the microporous channels of ZSM-5 zeolite. The minor differences in adsorption capacity of these three samples at low pressure demonstrate that all samples possess intact microporous channels, consistent with the results of XRD, N_2_ sorption and ^27^Al MAS NMR. However, as the relative pressure rises, the adsorption capacity of Na-Z5-0.15-25-140 increases sharply since it forms the abundant small mesopores in the nanocrystallite assembly, and K-Z5-0.15-25-140 with intracrystal mesopores only adsorbs *p*-xylene at higher pressure (*P*/*P*_0_ > 0.8). Therefore, both hierarchical ZSM-5 zeolites exhibit higher adsorption isotherms than pure microporous SC-Na-Z5 (Figure 7), indicating their higher adsorption capacity. As for *o*-xylene, the adsorption capacity of three samples is much lower than that of *p*-xylene, and the adsorption amount follows the order of Na-Z5-0.15-25-140 > K-Z5-0.15-25-140 > SC-Na-Z5 over the whole range of test pressure, which matches well with the trend of their catalyst activity and stability. Combined with the previous reaction data, it can be seen that the diffusion of the product (*p*-xylene) away from acid sites is similar in these three samples, but their secondary reactions of the product on the external surface probably determine the selectivity of the whole reaction. 

## 4. Conclusions

In this work, we successfully synthesized a series of zeolites with varied morphologies and mesostructures in a seed-assisted system by simply adjusting the basic parameters of zeolite synthesis (inorganic cation species, water-to-silicon ratio, alkalinity and temperature). Subsequently, we further explored the relationship between the synthesis parameters and the crystallization mechanisms by means of directional etching in NH_4_F or NaOH solutions, including the evolution of seed crystals, the development and assembly behavior of aluminosilicate species and the solution-phase process during zeolite growth and crystallization. In the K^+^ ion system, the seed crystals dissolve and transform into the structural species with the “memory” of original seeds to participate in the zeolite growth process under the action of K^+^; while in the Na^+^ ion system, the seed crystals tend to remain to guide the epitaxial growth of aluminosilicate species. Moreover, compared with K^+^ ions, the aluminosilicate species in the solution have more opportunities to form short-range ordered nanoparticles in the presence of Na^+^ ions. This also means that under the same alkali metal ion concentration, there will be more monomers and oligomers in the K^+^ ion system, which will have more classical crystallization processes. Then, we selected three typical ZSM-5 zeolites with diverse structures and the same particle size and acidity for the adsorption of the *p*/*o*-xylene and isomerization of *o*-xylene, indicating that different crystallization processes have a significant impact on the zeolite structure, thereby synergistically enhancing catalytic activity and shape selectivity.

## Figures and Tables

**Table 1:** Initial synthesis compositions or conditions and corresponding product phase in ions-mediated seed-assisted synthesis.

No.	Pre-Added Seeds *^a^*	M^+^ ion	H_2_O/SiO_2_ *^b^*	M_2_O/SiO_2_ *^b^*	*T*/°C *^b^*	Time/h *^b^*	Product Phase *^c^*
1	silicalite-1	Na^+^	15	0.15	140	96	MFI + Im.
2	silicalite-1	Na^+^	25	0.15	140	72	MFI
3	silicalite-1	Na^+^	50	0.15	140	72	MFI
4	silicalite-1	Na^+^	80	0.15	140	72	MFI
5	silicalite-1	K^+^	15	0.15	140	48	MFI
6	silicalite-1	K^+^	25	0.15	140	24	MFI
7	silicalite-1	K^+^	50	0.15	140	24	MFI
8	silicalite-1	K^+^	80	0.15	140	24	MFI
9	silicalite-1	Na^+^	25	0.08	140	96	MFI
10	silicalite-1	Na^+^	25	0.28	140	72	MFI
11	silicalite-1	K^+^	25	0.08	140	48	MFI
12	silicalite-1	K^+^	25	0.28	140	24	MFI
13	silicalite-1	Na^+^	25	0.15	120	96	MFI
14	silicalite-1	Na^+^	25	0.15	180	8	MFI
15	silicalite-1	K^+^	25	0.15	120	48	MFI
16	silicalite-1	K^+^	25	0.15	180	8	MFI
17	none	Na^+^	25	0.15	140	72	MFI + Im.
18	none	K^+^	25	0.15	140	72	Am.

*^a^* The silicalite-1 with a size of ca. 250 nm was used as seeds in general. *^b^* Other synthetic conditions are maintained at Al_2_O_3_/SiO_2_ = 0.0125 and time represents hydrothermal time. *^c^* Im. = impurities and Am. = amorphous phase.

## Data Availability

Data can be available upon request from the authors.

## Supplementary Materials

Supporting information can be downloaded at https://www.mdpi.com/article/10.3390/nano12091601/s1, Figure S1: XRD patterns of K-Z5-0.15-y-140 (y = 15/25/50/80) samples; Figure S2. The SEM images of the products synthesized in the K^+^ system with H_2_O/SiO_2_ = (A) 15, (B) 25, (C) 50 and (D) 80; Figure S3: XRD patterns of Na-Z5-0.15-y-140 (y = 15/25/50/80) samples; Figure S4: The SEM images of the products synthesized in the Na^+^ system with H_2_O/SiO_2_ = (A) 15, (B) 25, (C) 50 and (D) 80; Figure S5: XRD patterns of (A)K-Z5-x-25-140 (x = 0.08/0.15/0.28) and (B)Na-Z5-x-25-140 (x = 0.08/0.15/0.28) samples; Figure S6. XRD patterns of (A)K-Z5-0.15-25-*T* (*T* = 120/140/180) and (B) Na-Z5-0.15-25-*T* (*T* = 120/140/180) samples; Figure S7: XRD patterns of K-Z5-0.15-25-140 and Na-Z5-0.15-25-140 samples synthesized without seeds after 72 h of hydrothermal treatment; Figure S8: The TEM image of the SC-Na-Z5; Figure S9: The pore size distribution of the SC-Na-Z5, K-Z5-0.15-25-140 and Na-Z5-0.15-25-140 samples; Table S1: The textural properties of calcined Na-Z5-0.15-25-140, K-Z5-0.15-25-140 and SC-Na-Z5 products; Table S2: The acid amounts measured by NH_3_-TPD of H^+^-form Na-Z5-0.15-25-140, K-Z5-0.15-25-140 and SC-Na-Z5 products.
