# Supplementary material for: Facile Morphology and Porosity Regulation of Zeolite ZSM-5 Mesocrystals with Synergistically Enhanced Catalytic Activity and Shape Selectivity
Source: Nanomaterials (Basel). 2022 May 9;12(9):1601. doi: 10.3390/nano12091601 (PMC9105084; doi:10.3390/nano12091601)
Supplement: Supplementary file 1 [file nanomaterials-12-01601-s001.zip › nanomaterials-1703059-supplementary.pdf]

nanomaterials

*Supplementary Materials*

# Facile Morphology and Porosity Regulation of Zeolite ZSM-5 Mesocrystals with Synergistically Enhanced Catalytic Activity and Shape Selectivity

**Feng Lin [1,†], Zhaoqi Ye [1,†], Lingtao Kong [1], Peng Liu [2], Yahong Zhang [1], Hongbin Zhang [2,*] and Yi Tang [1,*]**

1 Department of Chemistry, Shanghai Key Laboratory of Molecular Catalysis and Innovative Materials, and Laboratory of Advanced Materials, Fudan University, Shanghai 200433, China; 20210220046@fudan.edu.cn (F.L.); 17110220035@fudan.edu.cn (Z.Y.); konglingtao@fudan.edu.cn (L.K.); zhangyh@fudan.edu.cn (Y.Z.)

2 Institute for Preservation and Conservation of Chinese Ancient Books, Fudan University Library, Fudan University, Shanghai 200433, China; liupengfdu@fudan.edu.cn

* Correspondence: zhanghongbin@fudan.edu.cn (H.Z.); yitang@fudan.edu.cn (Y.T.)

† These authors contributed equally to this work.

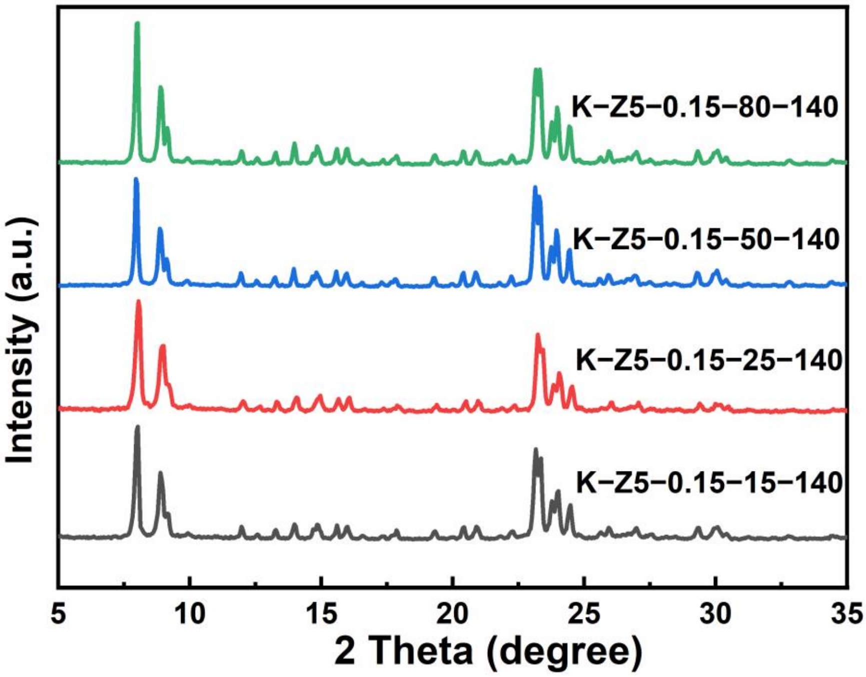


**Figure S1.** XRD patterns of K-Z5-0.15-y-140 (y = 15/25/50/80) samples.

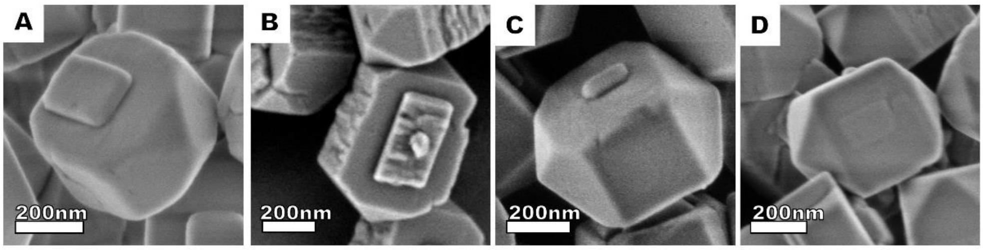


**Figure S2.** The SEM images of the products synthesized in the $K^+$ system with $H_2O/SiO_2$ = (**A**) 15, (**B**) 25, (**C**) 50 and (**D**) 80.

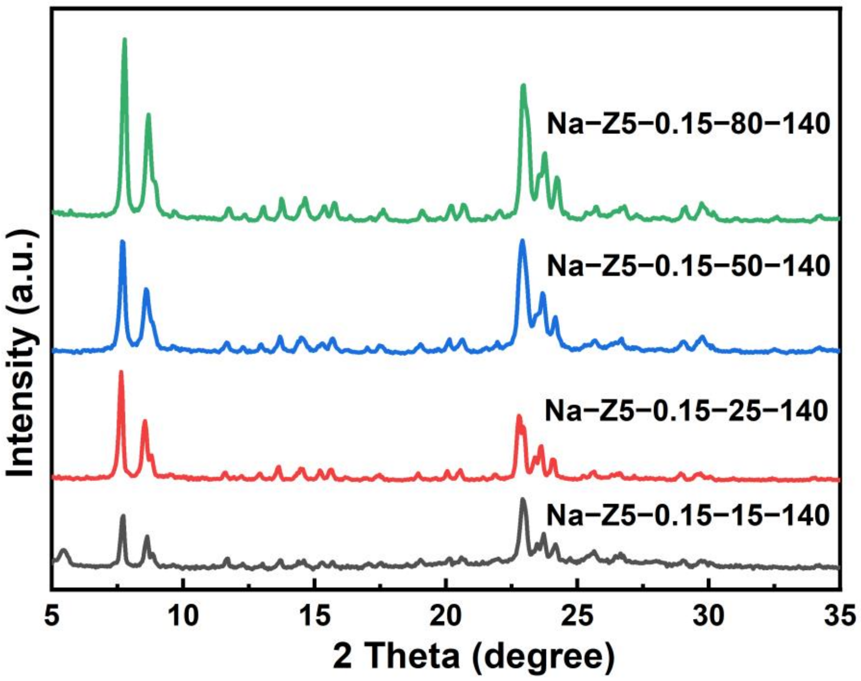


**Figure S3.** XRD patterns of Na-Z5-0.15-y-140 (y = 15/25/50/80) samples.

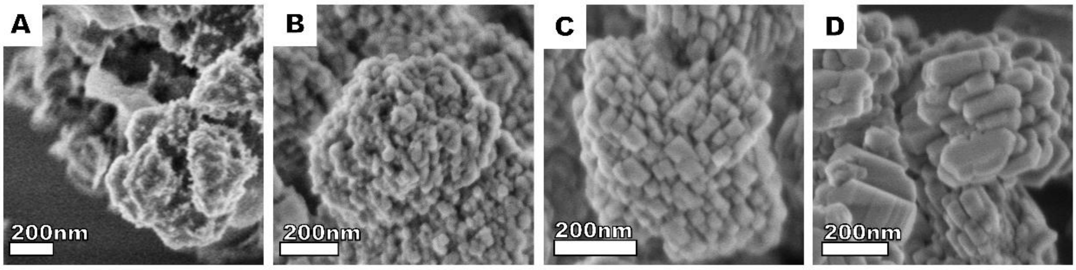


**Figure S4.** The SEM images of the products synthesized in the $Na^+$ system with $H_2O/SiO_2$ = (**A**) 15, (**B**) 25, (**C**) 50 and (**D**) 80.

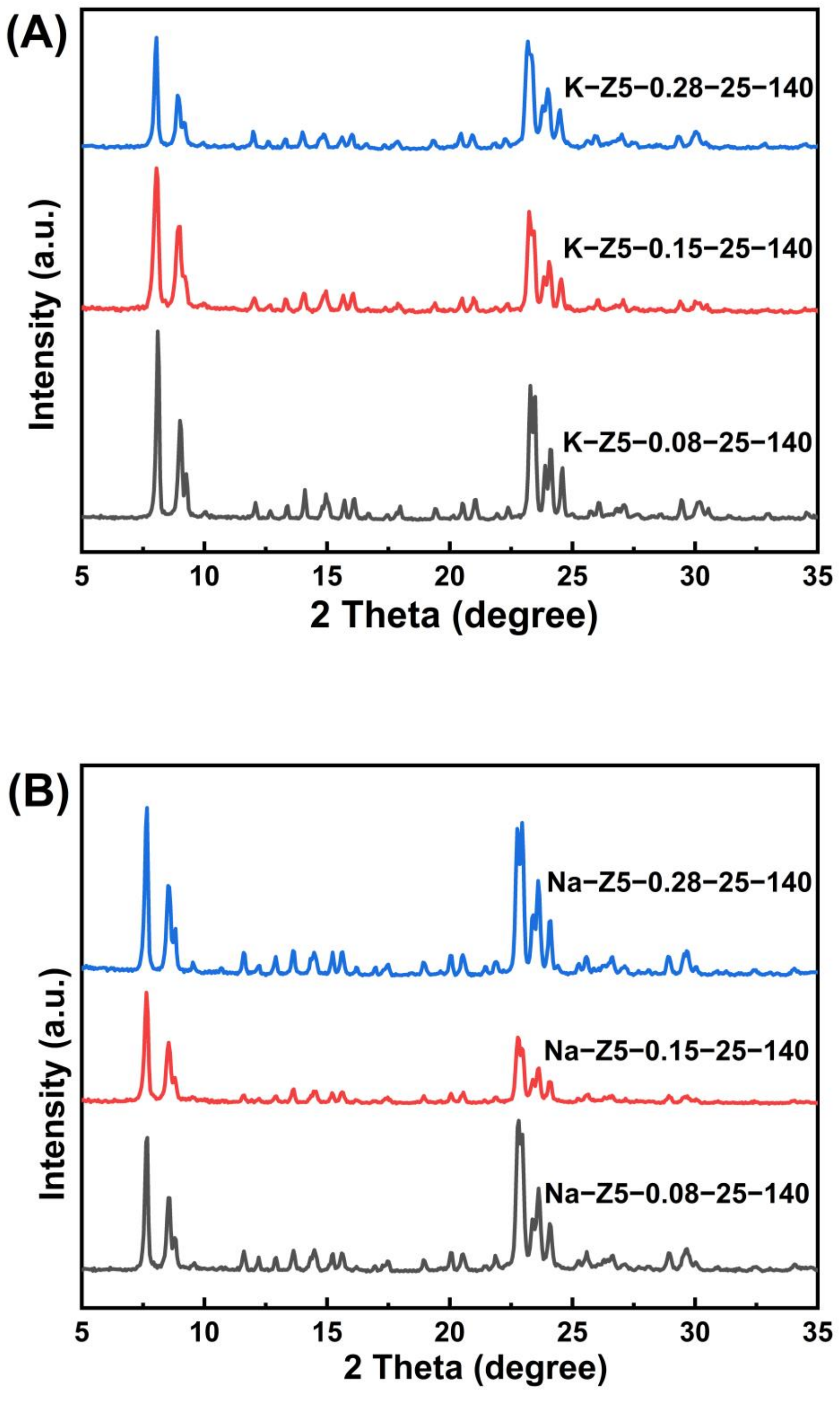


**Figure S5.** XRD patterns of (**A**)K-Z5-x-25-140 (x = 0.08/0.15/0.28) and (**B**)Na-Z5-x-25-140 (x = 0.08/0.15/0.28) samples.

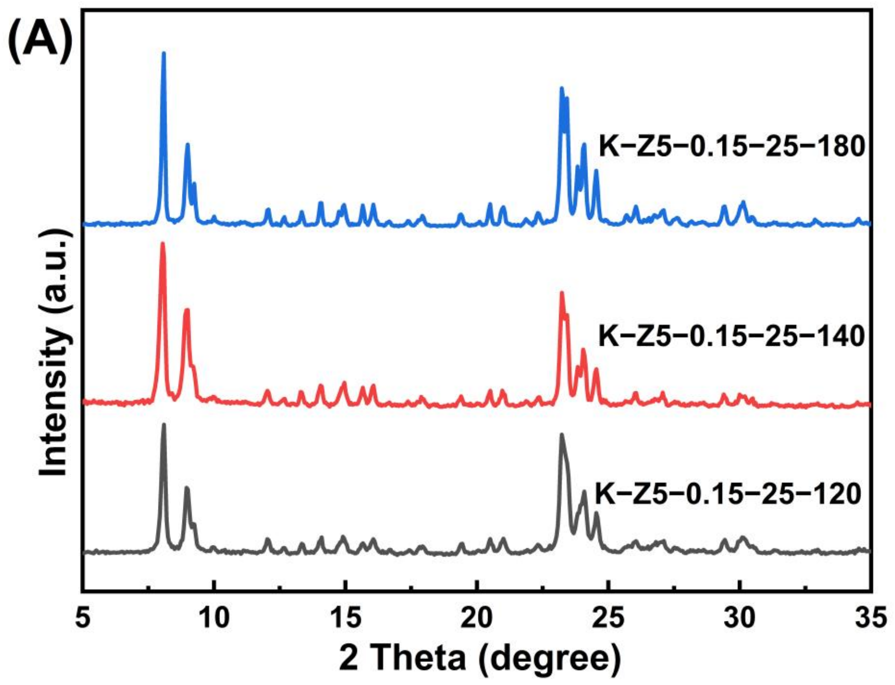


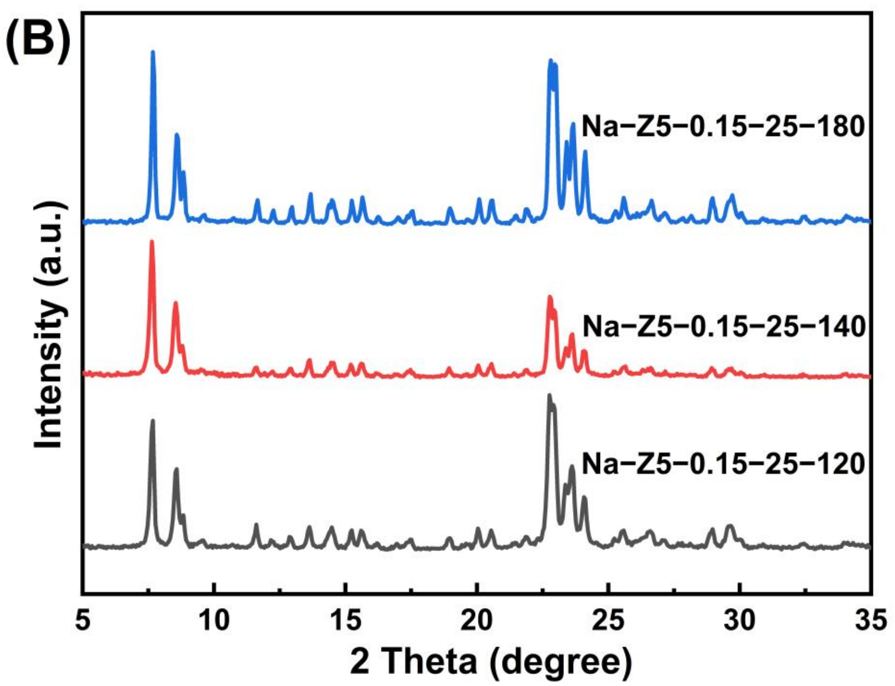


**Figure S6.** XRD patterns of (**A**)K-Z5-0.15-25-*T* (*T* = 120/140/180) and (**B**) Na-Z5-0.15-25-*T* (*T* = 120/140/180) samples.

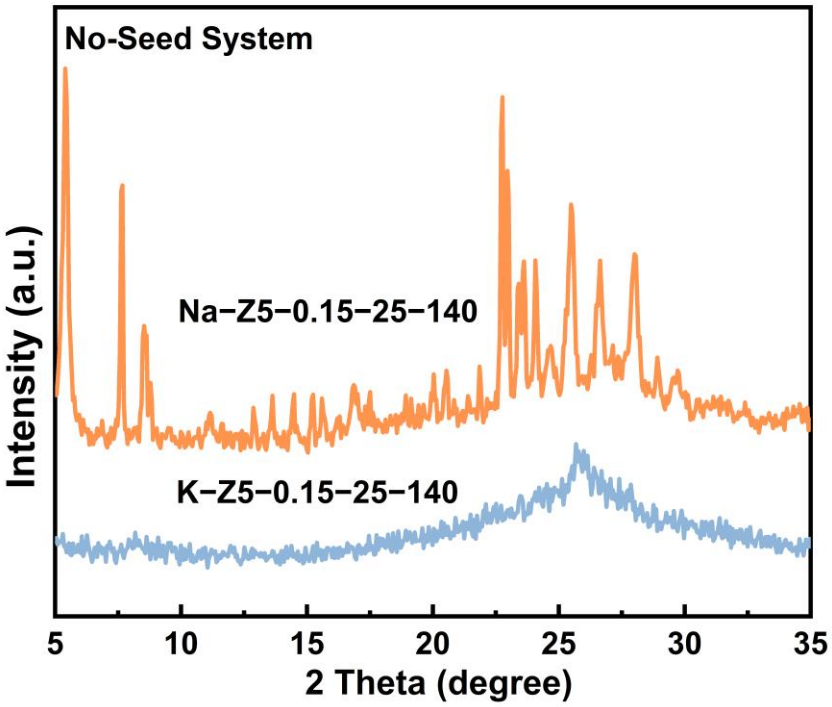


**Figure S7.** XRD patterns of K-Z5-0.15-25-140 and Na-Z5-0.15-25-140 samples synthesized without seeds after 72 hours of hydrothermal treatment.

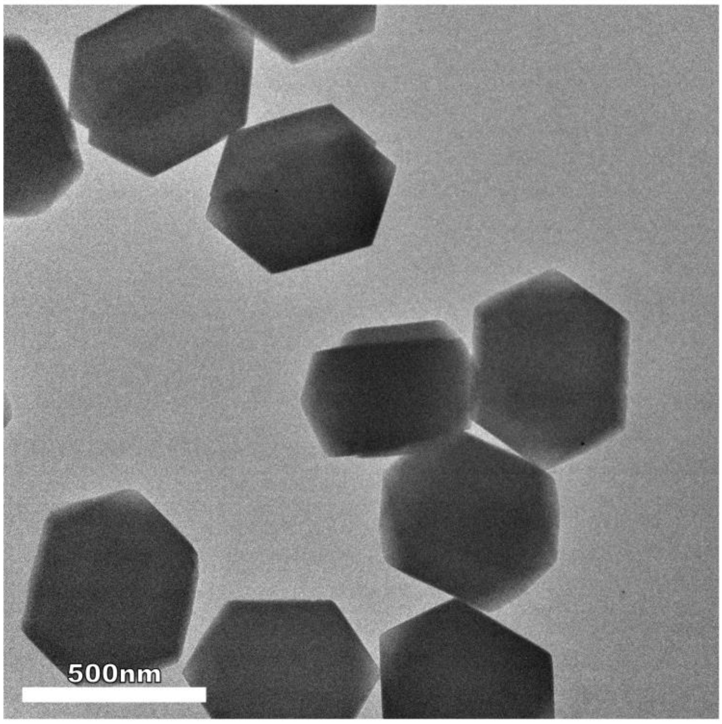


**Figure S8.** The TEM image of the SC-Na-Z5.

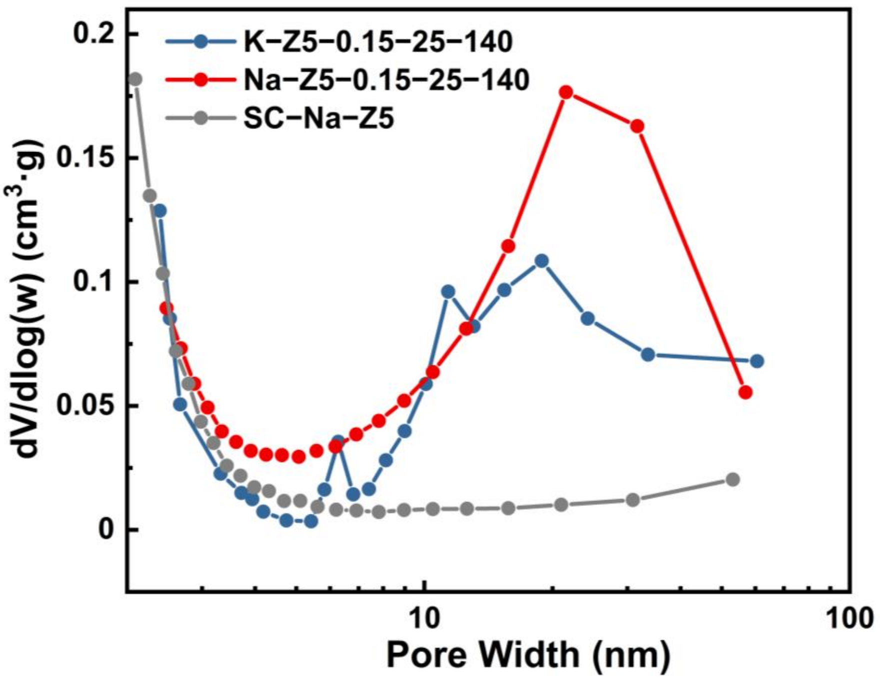


**Figure S9.** The pore size distribution of the SC-Na-Z5, K-Z5-0.15-25-140 and Na-Z5-0.15-25-140 samples.

**Table S1.** The textural properties of calcined Na-Z5-0.15-25-140, K-Z5-0.15-25-140 and SC-Na-Z5 products.

| Samples | $S_{BET}$ [a] ($m^2 \cdot g^{-1}$) | $S_{micro}$ [b] ($m^2 \cdot g^{-1}$) | $S_{ext}$ [b] ($m^2 \cdot g^{-1}$) | $V_{micro}$ [b] ($cm^3 \cdot g^{-1}$) | $V_{meso}$ [c] ($cm^3 \cdot g^{-1}$) |
|---|---|---|---|---|---|
| **Na-Z5-0.15-25-140** | 407 | 309 | 98 | 0.120 | 0.180 |
| **K-Z5-0.15-25-140** | 399 | 336 | 63 | 0.138 | 0.149 |
| **SC-Na-Z5** | 387 | 348 | 39 | 0.141 | 0.043 |

[a] determined by the relative pressure $P/P_0$ from 0.005 to 0.035. [b] by t-plot method. [c] using BJH method.

**Table S2.** The acid amounts measured by $NH_3$-TPD of $H^+$-form Na-Z5-0.15-25-140, K-Z5-0.15-25-140 and SC-Na-Z5 products.

| Samples | Weak acid ($\mu mol \cdot g^{-1}$) [a] | Strong acid ($\mu mol \cdot g^{-1}$) [b] |
|---|---|---|
| **Na-Z5-0.15-25-140** | 459 | 303 |
| **K-Z5-0.15-25-140** | 430 | 281 |
| **SC-Na-Z5** | 479 | 303 |

[a] The amounts of weak acid sites were measured by the amounts of $NH_3$ desorbed at 80–280 °C.

[b] The amounts of strong acid sites were measured by the amounts of $NH_3$ desorbed at 280–550 °C.
